# Coronary computed tomographic angiograph as gatekeeper?—The gate is wide open

**DOI:** 10.1007/s12471-021-01640-0

**Published:** 2021-10-22

**Authors:** R. Delewi, I. I. Tulevski

**Affiliations:** 1 Department of Cardiology, Amsterdam University Medical Centres, location Amsterdam Medical Centre, University of Amsterdam, Amsterdam, The Netherlands; 2 Cardiology Centres of the Netherlands, Amsterdam, The Netherlands

In the Netherlands, approximately 350,000 patients are referred to a cardiologist for a first evaluation of cardiac complaints. It is estimated that half of this population are assessed for chest pain. As such, there is an ongoing interest in the diagnostic workup of patients with chest pain and suspected coronary artery disease (CAD). In recent years, there have been important technical developments in the field of coronary computed tomographic angiography (CCTA). New trials have been published, and clinical guidelines have advocated a more important role for CCTA in clinical practice. Further advances in hardware and advances analytics will lead to a core role for CCTA at the centre of every clinical cardiovascular practice.

The SCOT-HEART (Scottish Computed Tomography of the Heart Trial) demonstrated the added value of CCTA to standard of care (which included an exercise electrocardiogram in most patients) [1]. The addition of CCTA clarified the diagnosis of angina due to epicardial coronary heart disease. In this trial, the need for further stress testing and invasive coronary angiography was reduced. More focused treatment regimens, as dictated by the cardiologist, were associated with a reduction in fatal and non-fatal myocardial infarction. This effect was largest when the National Institute for Health and Care Excellence criteria were added to the SCOT-HEART cohort, reflecting the patient with atypical and typical angina [2].

The large randomised controlled PROMISE (Prospective Multicenter Imaging Study for Evaluation of Chest Pain), which included over 10,000 patients with stable angina pectoris, demonstrated that CCTA was non-inferior to a functional testing approach (MRI, PET/SPECT or stress echocardiography) with respect to the composite endpoint of death, myocardial infarction, hospitalisation for unstable angina or major procedural complications [3]. However, the cost-effectiveness analysis of the PROMISE trial showed that CCTA results in lower costs than functional testing [4]. This is something to consider given the large number of patients who are evaluated for chest pain.

In this issue of the *Netherlands Heart Journal*, Boerhout et al. argue an even more dominant role for CCTA than used so far in patients with new-onset stable angina presenting at the outpatient clinic [5]. They demonstrate a helpful diagram for the evaluation of chest pain patients, which can be used in daily clinical practice. In fact, in their opinion paper, they propose CCTA as a gatekeeper for all these patients.

In the *Cambridge Dictionary*, a gatekeeper is described as ‘someone who has the power to decide who gets particular resources and opportunities, and who does not’. In the published flow chart, CCTA will dictate treatment strategy (including medication for primary prevention) and which patient should be referred for further additional noninvasive or invasive testing [5]. Indeed, subanalysis of the PROMISE trial showed a significant improvement of patient compliance to statin therapy in the CCTA arm regardless of CCTA outcome. The lower adherence to statins in the group guided by a functional test (86% in CCTA group vs 67% in functional group) was associated with a higher rate of major adverse cardiac events during the 2 to 5 years of follow-up in the functional group.

However, Boerhout and colleagues add an important notion by suggesting the use of CCTA for the individual patient. In the case of angina with no obstructive coronary artery disease, CCTA cannot discriminate between patients with and without functional disorders of the vessels. CCTA can merely be used to state that there is nonobstructive CAD. The evaluation of symptoms, including the information from CCTA, allows tailored medical treatment. This strategy may reduce the number of invasive procedures, both in nonobstructive and obstructive CAD, if invasive procedures are reserved for those patients who do not respond adequately to installed medical therapy. This means intracoronary function testing in nonobstructive CAD and revascularisation in obstructive CAD.

Therefore, the evaluation conducted by the physician remains the cornerstone of diagnosis and treatment in patients with chest pain and can be regarded as the real gatekeeper for patient management. We are lucky that this is the case, as it makes evaluation in a large number of patients with chest pain an interesting challenge for the medical detectives (MDs).
